# Dilemma in approach to stroke in sickle cell disease patient: A case report

**DOI:** 10.1097/MD.0000000000029131

**Published:** 2022-07-15

**Authors:** Lina Okar, Hadeel Ali Alzoubi, Safa Shukur Mahmud, Ahmed Elyas, Mohamed A. Yassin

**Affiliations:** a Department of Medical Education, Hamad Medical Corporation, Doha, Qatar; b Department of Pediatrics, Hamad Medical Corporation, Doha, Qatar; c Department of Cardiology, Hamad Medical Corporation, Doha, Qatar; d Department of Medical Oncology, Hematology Section, National Center for Cancer Care and Research, Hamad Medical Corporation, Doha, Qatar.

**Keywords:** CVA, PFO, sickle cell disease, stroke

## Abstract

**Introduction:**

Cerebrovascular accidents in sickle cell disease (SCD) patients carry a high socioeconomic impact and represent the most important cause of morbidity, neurological deficits, and impaired quality of life in SCD young population.

Patent foramen ovale (PFO) is prevalent in 25% of the general population and it is associated with ischemic stroke in the young population via paradoxical embolism, yet there are no specific guidelines to address how to manage SCD patients with PFO who suffer a stroke.

**Patient concerns and diagnosis:**

Here we present a young SCD patient, aged 24 years, who suffered a stroke in childhood and later was discovered to have a PFO on subsequent echocardiography. The patient has been receiving blood transfusion therapy since 3 years of age.

**Interventions and outcomes:**

No treatment was administered to the patient.

The intervention that was done was echocardiography with a bubble study to detect PFO.

**Conclusion:**

Recommendations need to be put in place regarding screening for PFO in patients with SCD, in addition to highlighting issues of whether screening needs to be done in patients who have not developed stroke, and if PFO were to be found, what would be the best management approach and how will prognosis be affected.

## 1. Introduction

Sickle cell disease (SCD) is an autosomal recessive disorder due to a mutation in hemoglobin S, which results in a deformity to the red blood cells, transforming them into sickle-shaped cells that do not function normally, especially in case of hypoxemia, leading to a wide variety of clinical presentations that increase the morbidity and mortality of the disease. SCD falls in high consideration among hemoglobinopathies due to its widespread prevalence in Sub-Saharan Africa, South–Central America, India, Middle East, and other Mediterranean countries. Among Middle Eastern countries, the prevalence was reported from Multiple regions as follows: Qatar 3.9%, Oman 3.8%, Bahrain 2.1%, United Arab Emirates 1.9%, Yemen 0.95%, and Saudi Arabia 0.01% to 0.10%.^[1]^

SCD has 2 genotypes: homogeneous HbSS, which represents about two-thirds of cases, and heterogenous HbSC, which has a milder presentation. Other variants also present include HbSD, HbSE, HbSO, etc or the beta-thalassemia variant. The main pathophysiology of the disease is the vaso-occlusive crisis and cerebrovascular events. SCD is a multiorgan disease; complications include anemia, acute chest syndrome, and splenic dysfunction.^[1]^

One of the most common complications is the neurologic ones. In children, SCD is categorized between the most common causes of stroke. Recently, studies in this field have focused more on the central nervous system injuries in the context of SCD, as well as primary and secondary prevention. Between 1970 and 2010, 75% of infarcts in adults and children with SCD were ischemic.^[2]^ Though it is very common, there is still no clear evidence regarding the best strategy for prevention in developing countries. The exact mechanism of stroke, whether ischemic or hemorrhagic, is still not clearly defined; however, risk factors were introduced, which include reduced oxygen content in patients with SCD, presence of cerebrovasculopathy, increased cerebral demand in the setting of acute febrile illness, the presence of other cardiovascular risk factors, prior cerebral infarcts, and finally the rapid increase in hemoglobin levels with autotransfusion or therapy. Neurological injuries that are most prevalent among adults include silent cerebral infarct, ischemic stroke, hemorrhagic stroke, and aneurysms.^[3]^

Due to the impact stroke has on SCD patients in terms of quality of life, disability, success in academics, and employment, screening is important. The National Heart, Lung, and Blood Institute released a guideline that recommends screening children from age 2 to 16 years with annual transcranial Doppler (TCD); however, studies have reported a low rate of screening. TCD is the major and only method to date that can identify SCD patients who are at risk of stroke.^[3]^ TCD detects the high flow velocity at cerebral arteries and results might be affected by hydroxyurea therapy.

## 2. Case report

A 24-year-old man from Oman with SCD and a history of stroke at the age of 3 years, currently on continuous blood transfusion every 4 weeks, presented to the hematology clinic. As a child and adolescent, he had infrequent painful crises (in the form of acute chest syndrome) and liver siderosis as complications associated with the underlying hemoglobinopathy. He was on folic acid, hydroxyurea, and deferoxamine. His family history, social history, and review of systems were otherwise unremarkable. He had a history of stroke at the age of 3 years which presented in the form of right-sided paralysis that resolved after a short period of time. The computed tomography report for the event, the transcranial ultrasound (US) Doppler, and transthoracic echocardiogram (TTE) were not available for review; however, his TTE and carotid US Doppler performed a few years after the event in 2006 were both normal. Since then, he has been on chelation therapy and blood transfusion every 4 weeks as secondary prevention for stroke.

He presented at the age of 19 years with new-onset left-hand tremor, mainly when holding objects. There was no history of head trauma reported, no history of resting tremor, and the tremor did not interfere with his activities of daily living or writing despite being left-handed. Subsequently, magnetic resonance imaging was done and was significant for encephalomalacia on the left side, which could reflect the previous ischemic event he had at the age of 3 years. No new infarcts were appreciated and transcranial US Doppler was not recommended at this age. His regular follow-up echocardiograms were reported as normal at that time.

At the age of 22 years, he had 2 episodes of acute chest syndrome, one of which was complicated by hospital-acquired pneumonia with prolonged hospitalization. Later he was admitted with a 2-day history of bilateral upper limb pain associated with left hip and thigh pain. A complete blood count was comparable to his baseline with a hemoglobin of 9.8 g/dL, hematocrit of 30.6%, and reticulocyte count of 8.7%. Right arm Doppler US did not find evidence of deep vein thrombosis (DVT), but the cephalic vein showed thrombophlebitis changes. A Doppler ultrasound of the lower extremities showed no evidence of DVT (Table 1).

**Figure 1.**
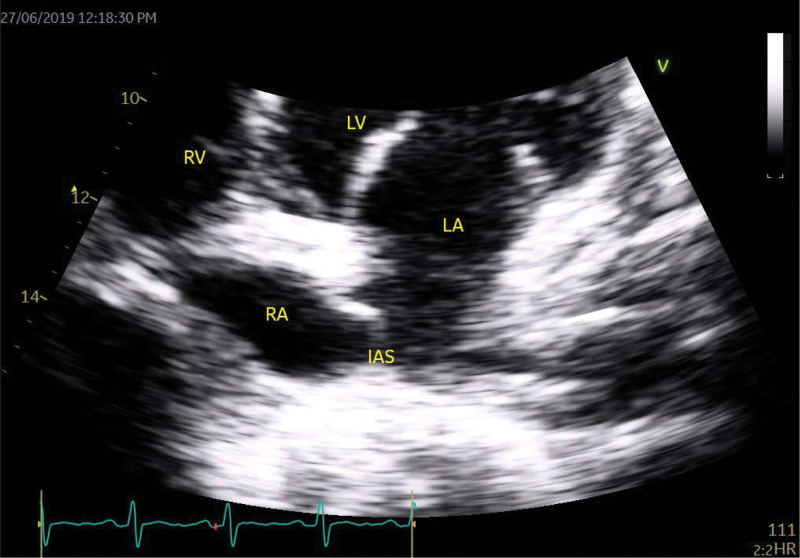
Four-chamber echocardiography view showing redundant interatrial septum frequently encountered with PFO. IAS = interatrial septum, LA = left atrium, LV = left ventricular, PFO = patent foramen oval, RA = right atrium, RV = right ventricular.

**Figure 2.**
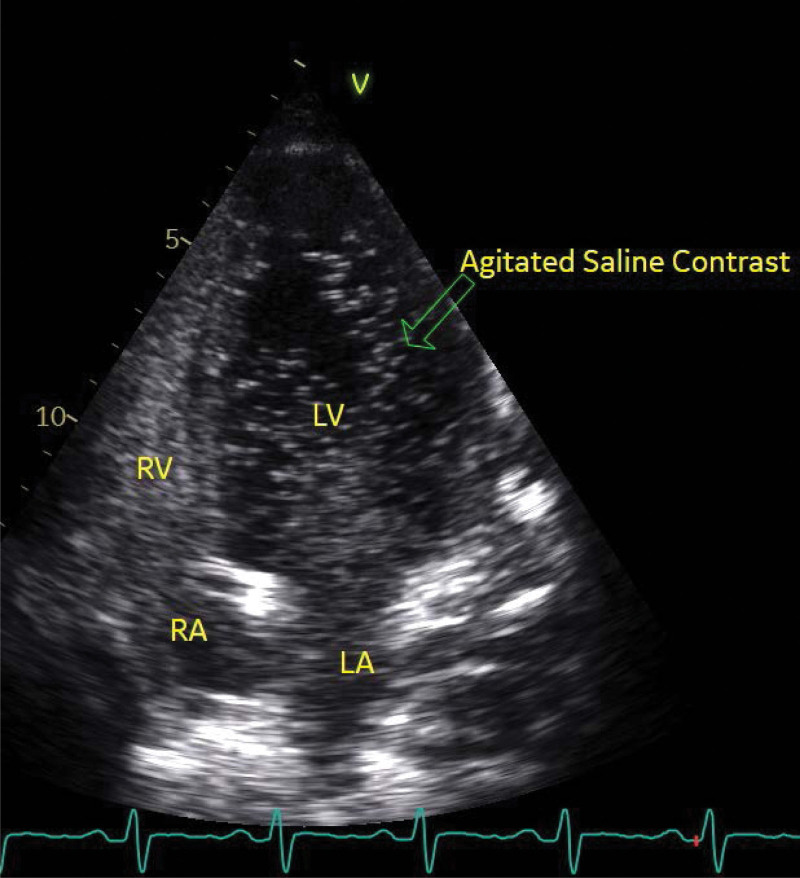
Four-chamber echocardiography view, which shows evidence of early bubble contrast passage through interatrial septum indicating the presence of patent foramen oval. LA = left atrium, LV = left ventricular, RA = right atrium, RV = right ventricular.

TTE was done to rule out infective endocarditis and showed normal global systolic left ventricular function (ejection fraction 55%) with evidence of a large patent foramen ovale (PFO), following agitated saline contrast injection (Figs. 1 and 2). There were no regional wall motion abnormalities and no evidence of infective endocarditis. He was managed as a sickle pain crisis and referred to cardiology services for his newly discovered PFO.

He continued to have 2 acute pain crises requiring admissions in the following 2 years. He was referred to a cardiology clinic for possible closure of his large PFO, which was postponed due to the coronavirus disease 2019 pandemic.

The patient has been on blood transfusion for almost 20 years after the infarct he had when he was 3 years of age. It is unclear whether his previous infarct was attributed to his hemoglobinopathy or to the prominent PFO detected later in his life, as there was a lack of investigations when he had the stroke.

## 3. Discussion

PFO is common in the general population as up to 25% of adults have it.^[4]^ It is well known that the presence of PFO increases the risk of cryptogenic stroke. Management of PFO is either by device closure or medical treatment alone. Closure is superior to medical management, and it is indicated in PFO patients <60 years of age with embolic stroke and no other identifiable cause of stroke.^[4]^ Many factors increase the risk of stroke in patients with SCD along with PFO, which are as follow: acute painful crisis, which increases intrathoracic pressure that leads to enlargement in the shunt; hypercoagulable state; prevalence of pulmonary hypertension; and endothelial dysfunction.^[5]^

However, there is no specific guidance regarding the best way to address PFO among SCD patients who suffer a stroke and most of the evidence in these patients comes from case reports or small studies. One pilot study in 2016 revealed that the prevalence of PFOs in adults with SCD and stroke of 33% (5/15) is quite similar to PFOs in the general population (24%) with similar prevalence in adults and children and showed that HbSS was the most common genotype with a high prevalence of DVT in these patients.^[6]^ In another study in children with SCD, 25% (10/40) of children with stroke were identified to have PFOs.^[7]^ Most of the few reported case reports were SCD cases with HbSS and presented as embolic stroke that occurred in children or young adults.^[6–8]^ Percutaneous closure of the PFO was considered in SCD patients <60 years of age with a history of recurrent ischemic strokes, as they are considered a high-risk group for recurrence.

Management of cerebrovascular events in the SCD population includes primary and secondary prevention. In primary prevention, only 1 strategy gained approval which is chronic blood transfusion and controlling risk factors. A study called Stroke Prevention Trial in Sickle Cell Anemia (STOP), which included 2000 children with SCD, concluded the TCD and blood transfusion role in reducing stroke risk. Another study (TCD with transfusions changing to hydroxyurea) evaluating the role of hydroxyurea in primary prevention suggested that the maximum tolerated dose of hydroxyurea can substitute for regular blood transfusion. However, though there is uncertainty about the primary prevention of ischemic stroke in SCD patients, the aim according to American Society of Hematology is to always keep hemoglobin >9 g/dL and hemoglobin S <30%. The role of anticoagulation and antiplatelet therapy in the prevention of stroke in SCD patients has not been established yet. Recommendations currently advise aspirin, warfarin, or heparin in the secondary prevention only if embolic stroke occurs with evidence of thrombosis. Recent European statements for patients with PFO suggest closing it in some situations like hypercoagulable state with a high risk of bleeding on medical therapy. If this statement holds true for SCD patients, then it might be beneficial to close the PFO, but proof of whether this will prevent further cerebrovascular events has not yet been documented in the literature.^[5]^

Our patient is a case of SCD with HbSS who had a stroke at the age of 3 years and in whom no TCD was done for him at the time. When he presented to the hematology clinic, he was already on continuous blood transfusion as secondary prevention for stroke. PFO was detected incidentally on echocardiogram after the bubble study though it was done multiple times before. There are 2 dilemmas in this situation; the first is identifying the cause of the stroke, whether it is SCD or PFO, as SCD requires aggressive blood exchange or continuous blood transfusion, which is a point of great value for developing countries, while on the other hand, PFO is managed by device closure. The second dilemma is the guideline statement about PFO closure, which states that the diagnosis of cryptogenic stroke is considered after exclusion of other potential stroke mechanisms, including hypercoagulable state that includes SCD. Some important questions to consider here: Is there a need to screen for PFO in patients with SCD even though no stroke has developed? And if PFO was found, what is the best approach to management and how will the prognosis be affected. Moreover, PFO device closure needs to be investigated in the pediatric population as there are limited data on children.

Although the benefit of PFO device closure is uncertain in SCD, a recent review suggested considering device closure over a conservative approach because of the pathophysiology of the SCD and the increased endothoracic pressures during a crisis.^[8]^

## 4. Conclusion

SCD is a multiorgan disease, which can lead to debilitating complications owing to its pathophysiology. Among the most common complications are the neurologic ones. In children and young adults, SCD is categorized between the most common causes of stroke; thus, this leads physicians to usually assume that the stroke is in the context of SCD, which might deviate them from considering other possibilities, like the presence of PFO, a well-known cause of cryptogenic stroke. In this case report, we highlight the dilemma regarding the need for screening for PFO in patients with SCD. This is because the presence of another cause of stroke might alter the management approach of patients and might help avoid unnecessary transfusions. Management of PFO is either by device closure or medical therapy. However, no specific guidance regarding the best way to manage PFO among SCD and stroke patients is present to date, and there are no recommendations to do investigations early in the course of the disease to detect the presence of PFO and no evidence-based management strategies.

To conclude, due to the impact stroke has on patients in terms of quality of life, recommendations need to be put in place regarding screening for PFO in patients with SCD, in addition to highlighting issues of whether screening needs to be done in patients who have not developed stroke, and if PFO were to be found, what would be the best management approach and how will prognosis be affected.

**Table 1:** Details of the imaging and laboratory investigations.

**Investigations**	
MRI cardiac and hepatic iron load	Very severe liver siderosis noted and no evidence of significant myocardial siderosis.
MRI head 2017	Dilated collateral arteries in the basal ganglia, adjacent deep cerebral white matter and basal cisterns. Small left frontal subcortical area of cystic encephalomalacia. Bilateral few cerebral white matter abnormal foci for clinical significance.
Upper limb Doppler US 2019	No evidence of DVT. Cephalic vein shows thrombophlebitic changes.
TTE 2019	Normal global systolic LV function (EF 55%). No regional wall motion abnormality. Evidence of a large patent foramen ovale. No evidence of infective endocarditis.
Blood tests	
ABO	Group B +
Hemoglobin (13–17 g/dL)	9.8
RBC (4.5–5.5 × 10^6^)	3.6
WBC (4–10 × 10^3^/µL)	13.6
ANC (2.0–7.0 × 10^3^/µL)	9.0
PLT (150–400 × 10^3^/µL)	463
Reticulocyte (0.2%–2.5%)	1.1
Lymphocytes count (1–3 × 10^3^/µL)	3.4
Ferritin (8–252 µg/L)	70150
CRP (0–5 µg/L)	297
D-dimer (0.00–0.4 µg/L)	
LDH (135–214 U/L)	
Renal function tests (urea/Cr) (2.1–8.8 mmol/L)/(44–80 µmol/L)	Urea: 4.5 Cr: 46
ALT/AST (0–33 U/L)/(0–32 U/L)	ALT: 25.9 AST: 29
TB (0–21 µmol/L)	96
Albumin (35–50 g/L)	34
G6PD	Normal
Hemoglobin electrophoresis	Hgb A: 13.8 Hgb A2: 4.7 Hgb F: 0.8 Hgb S: 80.7
CK (39–308 U/L)	16

ALT = alanine transaminase, ANC = absolute neutrophil count, AST = aspartate transaminase, CK = creatinine kinase, Cr = creatinine, CRP = C-reactive protein, DVT = deep vein thrombosis, EF = ejection fraction, G6PD = glucose-6-phosphate dehydrogenase, LDH = lactate dehydrogenase, LV = left ventricular, MRI = magnetic resonance imaging, PLT = platelet, RBC = red blood cell, TB = total bilirubin, TTE = transthoracic echocardiography, US = ultrasound, WBC = white blood cell.

## Acknowledgments

Open access fees offered by Qatar National Library (QNL).

## Author contributions

All authors contributed equally to writing the manuscript.

## References

[1] Wali Y Kini V Yassin MA. Distribution of sickle cell disease and assessment of risk factors based on transcranial Doppler values in the Gulf region. Hematology. 2020;25:55–62. 31983291 10.1080/16078454.2020.1714113

[2] Debaun MR Kirkham FJ. Central nervous system complications and management in sickle cell disease. Blood. 2016;127:829–38 26758917 10.1182/blood-2015-09-618579

[3] Reeves SL Madden B Freed GL. Transcranial Doppler Screening Among Children and Adolescents With Sickle Cell Anemia. JAMA Pediatr. 2016;170:550–56. 27064406 10.1001/jamapediatrics.2015.4859 PMC7111507

[4] Messé SR Gronseth GS Kent DM. Practice advisory update summary: patent foramen ovale and secondary stroke prevention: report of the Guideline Subcommittee of the American Academy of Neurology. Neurology. 2020;94:876–85 32350058 10.1212/WNL.0000000000009443 PMC7526671

[5] Aggeli C Polytarchou K Dimitroglou Y. Stroke and presence of patent foramen ovale in sickle cell disease. J Thromb Thrombolysis. 2021;52:889–97. 33638018 10.1007/s11239-021-02398-3 PMC7909731

[6] Razdan S Strouse JJ Reddy A. Patent foramen ovale in adults with sickle cell disease and stroke. Am J Hematol. 2016;91:E358–60 27253454 10.1002/ajh.24440

[7] Dowling MM Lee N Quinn CT. Prevalence of intracardiac shunting in children with sickle cell disease and stroke. J Pediatr. 2010;156:645–50. 20022343 10.1016/j.jpeds.2009.10.012 PMC4250927

[8] Galadanci NA Johnson W Carson A. Association between patent foramen ovale and overt ischemic stroke in children with sickle cell disease. Front Neurol. 2021;12:761443. 34966346 10.3389/fneur.2021.761443 PMC8710657

